# Supplementary material for: The Role of the ALDH Family in Predicting Prognosis and Therapy Response in Pancreatic Cancer
Source: Biomedicines. 2025 Aug 19;13(8):2018. doi: 10.3390/biomedicines13082018 (PMC12384013; doi:10.3390/biomedicines13082018)
Supplement: Supplementary file 1 [file biomedicines-13-02018-s001.zip › biomedicines-3777599-supplementary.pdf]

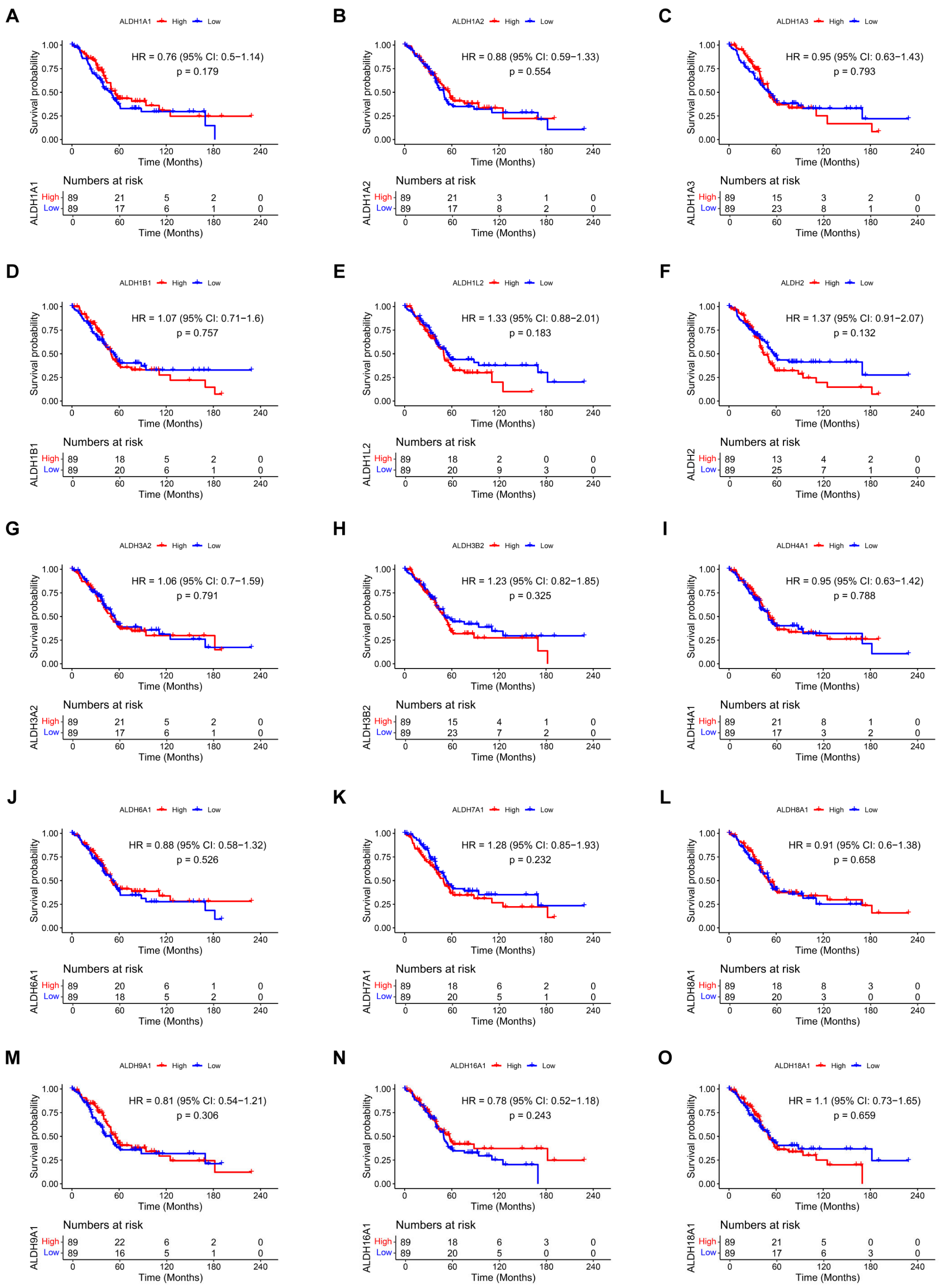

Figure S1. Kaplan-Meier curves of PAAD patients with above or below median expression levels of *ALDH1A1* (**A**), *ALDH1A2* (**B**), *ALDH1A3* (**C**), *ALDH1B1* (**D**), *ALDH1L2* (**E**), *ALDH2* (**F**), *ALDH3A2* (**G**), *ALDH3B2* (**H**), *ALDH4A1* (**I**), *ALDH6A1* (**J**), *ALDH7A1* (**K**), *ALDH8A1* (**L**), *ALDH9A1* (**M**), *ALDH16A1* (**N**), *ALDH18A1* (**O**) in PAAD.

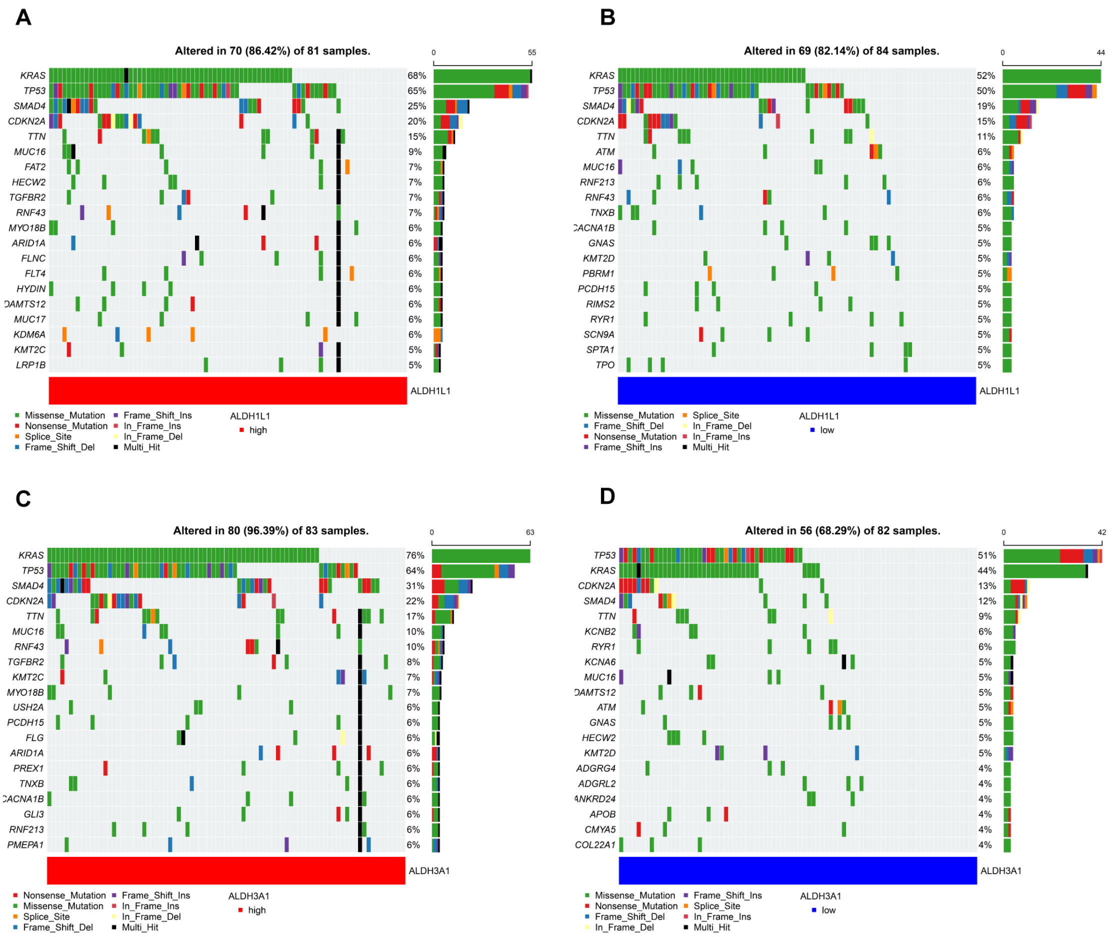

Figure S2. Estimation tumor mutational burden (TMB) in the ALDHs group. Analysis of gene mutation frequencies in the *ALDH1L1* (**A**) high- and (**B**) low-group. Analysis of gene mutation frequencies in the *ALDH3A1* (**C**) high - and (**D**) low- group.

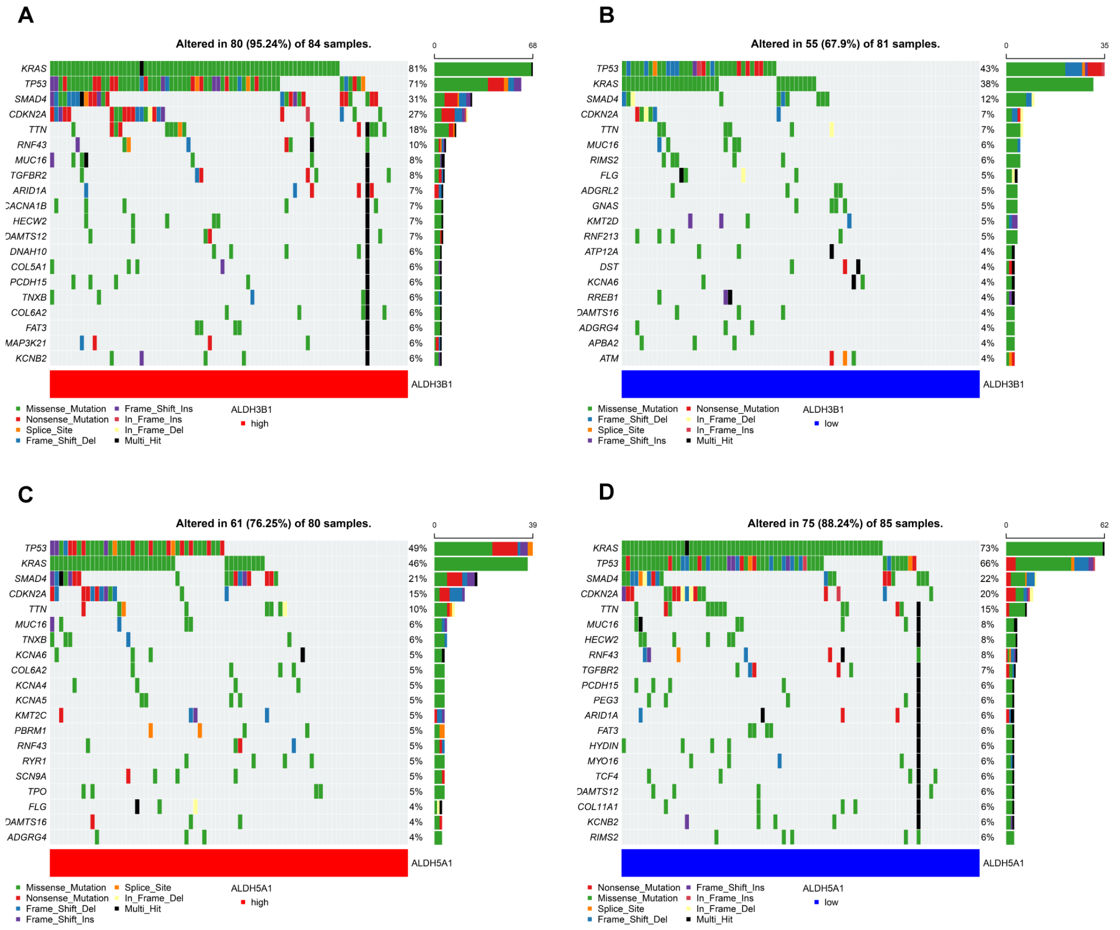

Figure S3. Estimation tumor mutational burden (TMB) in the ALDHs group. Analysis of gene mutation frequencies in the *ALDH3B1* (**A**) high- and (**B**) low-group. Analysis of gene mutation frequencies in the *ALDH5A1* (**C**) high - and (**D**) low- group.

Table S1. clinicopathological parameters of patients with pancreatic cancer in TCGA databases.

Covariates	Type	Total(n)	ALDH1L1 (high low)	ALDH3A1 (high low)	ALDH3B1 (high low)	ALDH5A1 (high low)
			P value	*P* value	*P* value	*P* value
Age			*0.88*	*0.0043*	*0.65*	*0.29*
	≤65	94	48 46	37 57	45 49	51 43
	>65	84	41 43	52 32	44 40	38 46
Gender			*0.65*	*0.65*	*0.097*	*1*
	Male	98	51 47	47 51	55 43	49 49
	Female	80	38 42	42 38	34 46	40 40
T Stage			*0.43*	*0.21*	*0.23*	*0.046*
	T1	7	5 2	3 4	4 3	6 1
	T2	24	11 13	8 16	8 16	14 10
	T3	142	72 70	76 66	75 67	67 75
	T4	3	1 2	2 1	2 1	0 3
	unknown	2	0 2	0 2	0 2	0 2
N Stage			*0.42*	*0.39*	*0.88*	*0.89*
	N0	49	21 28	25 24	24 25	24 25
	N1	124	66 58	63 61	63 61	62 62
	unknown	5	2 3	1 4	2 3	3 2
M Stage			*0.051*	*0.33*	*0.83*	*0.08*
	M0	80	48 32	36 44	38 42	34 46
	M1	4	2 2	3 1	2 2	1 3
	unknown	94	39 55	50 44	49 45	54 40
TNM Stage			*0.07*	*0.38*	*0.09*	*0.15*
	I	21	6 15	7 14	6 15	14 7
	II	147	80 67	76 71	79 68	72 75
	III	3	1 2	2 1	2 1	0 3
	IV	4	2 2	3 1	2 2	1 3
	unknown	3	0 3	1 2	0 3	2 1
